# Transcranial Doppler in Dementia: A Promising Tool for Early Detection and Subtyping

**DOI:** 10.7759/cureus.91973

**Published:** 2025-09-10

**Authors:** Baikuntha Panigrahi, Pradosh Kumar Sarangi

**Affiliations:** 1 Neurology, All India Institute of Medical Sciences, Deoghar, IND; 2 Radiodiagnosis, All India Institute of Medical Sciences, Deoghar, IND

**Keywords:** alzheimer's disease, dementia, dementia with lewy bodies, parkinson’s disease, substantia nigra, transcranial doppler, transcranial ultrasound

## Abstract

Dementia remains a leading cause of morbidity among older adults, and early diagnosis is crucial for timely intervention, improved outcomes, and appropriate care planning. While advanced neuroimaging techniques are considered the gold standard for early detection and subtyping, their limited availability in many regions underscores the need for accessible alternatives. Transcranial Doppler (TCD) and transcranial sonography (TCS) offer low-cost, noninvasive, and portable approaches to assess cerebral hemodynamics and structural brain changes. As evidence continues to evolve, these sonographic tools hold promise as complementary approaches to conventional imaging, particularly where advanced modalities are unavailable.

## Editorial

Alzheimer’s disease (AD) and other dementias represent the second leading cause of disability-adjusted life years (DALYs) among adults aged 60-79 years, accounting for 1504.2 (746.6-3345.6) per 100,000 people [1]. Early diagnosis is critical to improving care and outcomes. Timely interventions, including disease-specific therapies and advanced care planning, can reduce both morbidity and mortality. Early subtyping also allows prompt identification of reversible causes, which can prevent irreversible cerebral atrophy. Currently, early dementia detection relies heavily on advanced neuroimaging and nuclear medicine techniques, which are often inaccessible in resource-limited settings. In such contexts, transcranial Doppler (TCD), a noninvasive, low-cost, and widely available tool, offers a promising alternative. This editorial examines the potential role of transcranial sonography (TCS), including Doppler, in screening and subtyping early dementia and discusses key limitations in interpreting emerging evidence on its clinical utility.

Understanding TCD: principles and utility

TCD uses a much lower frequency phased array probe (2-2.5 MHz) to increase penetration and reduce the attenuation by bony structures. Four windows (transtemporal, transorbital, suboccipital, submandibular) allow insonation of the brain and its vessels. The various windows and their insonation depths are shown in Table 1.

**Table 1:** Acoustic windows, target vessels, insonation depths, and flow directions of major cerebral arteries MCA: middle cerebral artery; ACA: anterior cerebral artery; PCA: posterior cerebral artery; VA: vertebral artery; BA: basilar artery; OA: ophthalmic artery

Acoustic window	Target	Depth (mm)	Flow direction relative to probe	Mean flow velocity (cm/s)
Temporal	MCA	35-60	Toward	46-86
	ACA	60-75	Away	41-76
	PCA	60-75	Toward	33-64
Suboccipital	VA	45-74	Away	27-55
	BA	70-120	Away	30-57
Orbital	OA	40-50	Toward	16-26

This technique enables real-time monitoring of blood flow within the brain’s major arteries. It not only tracks the velocity of the blood but also provides insight into resistance in the downstream vessels by calculating indices like the resistivity index (RI) and pulsatility index (PI). One of its key strengths is its ability to capture rapid changes in blood flow with high temporal precision [2]. The TCD allows both structural and vascular insights into the brain, which makes it an invaluable tool for dementia diagnosis and subtyping.

Vascular insights in dementia: the role of Doppler

A systematic review found that individuals with AD and vascular dementia (VaD) exhibited slower cerebral blood flow velocities (CBv) and elevated PIs relative to cognitively normal controls, with PI being more prominently increased in VaD, suggesting increased vascular resistance and reduced vascular compliance [3]. However, it remains unclear whether these changes are a cause or consequence of the underlying neurodegenerative process. Interpretation of these findings is limited by the presence of multiple confounding factors commonly seen in elderly populations. For instance, an 80-year-old male patient with comorbid conditions such as diabetes, hypertension, dyslipidemia, and obstructive sleep apnea, all increasing the risk of small vessel disease, may show elevated PI values similar to those observed in AD. In this context, establishing age-adjusted reference cut-offs may offer more clinical utility than merely noting associations with altered PI values.

Since all included studies were observational in nature, they are inherently subject to biases such as selection bias and measurement bias, particularly given the operator-dependent nature of TCD. It is therefore challenging to determine whether the observed relationships are truly reflective of disease mechanisms or are confounded by external factors. Additionally, the small sample sizes (range: 17-194) in many of these studies limit the generalizability of their findings.

The utility of TCD in differentiating dementia subtypes also appears limited, as both AD and VaD consistently show reduced CBv and elevated PI. Although studies suggest that the increase in PI tends to be greater in VaD than in AD, this distinction lacks practical value due to the absence of well-defined absolute cut-offs for differentiation [4]. Also, individuals with mild cognitive impairment (MCI) exhibit reduced CBv compared to cognitively normal controls. How this mere nonspecific reduction in CBv without any impairment of dynamic autoregulation in MCI is currently unclear.

Better structural insights: TCS of the brain parenchyma

TCS, particularly B-mode imaging, allows visualization of brain structures like the medial temporal lobe (MTL), third ventricle (TV), and substantia nigra (SN). This modality could have utility in subtyping, along with the clinical picture (Table 2).

**Table 2:** Key TCS features of neurodegenerative diseases and their diagnostic utility MTL: medial temporal lobe; PPV: positive predictive value; NPV: negative predictive value; PSP-RS: progressive supranuclear palsy-Richardson syndrome; PSP-P: progressive supranuclear palsy-Parkinsonism

Neurodegenerative disease	Key TCS features	Diagnostic utility
Dementia with Lewy bodies	Marked SN hyperechogenicity + asymmetry index < 1.15	PPV and NPV=85.7%
Parkinson’s disease ± dementia	SN hyperechogenicity in ~33%; typically asymmetric	Differentiates from DLB based on symmetry
Multiple system atrophy-Parkinsonian type	Normal SN + marked lentiform nucleus hyperechogenicity	PPV=96% for MSA diagnosis
Progressive supranuclear palsy	Third ventricle >10 mm (PSP-RS), SN hyperechogenicity (PSP-P)	Helps subtype PSP-RS vs PSP-P
Corticobasal degeneration	Bilateral SN hyperechogenicity; normal third ventricle	Differentiates from PSP
Alzheimer’s disease	MTL atrophy	Supports AD diagnosis; MTL atrophy: sensitivity-83%, specificity-76%
Amyotrophic lateral sclerosis	SN hyperechogenicity (48-70%); enlarged third ventricle	Correlates with disease features; not specific
Creutzfeldt-Jakob disease	Bilateral lentiform hyperechogenicity; normal SN	May precede MRI/EEG changes

The asymmetry index is determined by dividing the larger SN area by the smaller SN area. In dementia with Lewy bodies (DLB), the combination of marked SN hyperechogenicity and an asymmetry index of the echogenic areas below 1.15 offers both a positive and negative predictive value of 85.7%, making it a promising marker for diagnosis [4]. In Parkinson’s disease with dementia and without dementia, about one-third of patients exhibit SN hyperechogenicity, typically with an asymmetric pattern, in contrast to the symmetrical echogenicity in DLB. 

Among other atypical Parkinsonian syndromes, TCS helps distinguish subtypes: in MSA-P, a combination of normal SN echogenicity and marked hyperechogenicity of the lentiform nucleus yields a 96% positive predictive value. In progressive supranuclear palsy (PSP), third ventricle enlargement (>10 mm) and lentiform nucleus hyperechogenicity are common in the Richardson subtype, whereas SN hyperechogenicity is more typical of PSP-Parkinsonism. Meanwhile, corticobasal degeneration often presents with bilateral SN hyperechogenicity but typically has a normal-sized third ventricle, helping to differentiate it from PSP [4].

In AD, most patients show either normal or only moderately increased SN echogenicity, with none displaying bilateral marked changes. The TCS can detect MTL atrophy with a sensitivity of 83% and specificity of 76% comparable to MRI, supporting its utility in suspected AD cases.

Amyotrophic lateral sclerosis (ALS) often presents with SN hyperechogenicity in 48-70% of patients and noticeable third ventricle enlargement. While there's no established TCS profile for frontotemporal dementia (FTD), extrapyramidal signs and basal ganglia changes seen in ALS may also appear in FTD, suggesting overlapping phenotypes.

In Creutzfeldt-Jakob disease (CJD), TCS can detect bilateral lentiform nucleus hyperechogenicity, sometimes even before abnormalities appear on MRI or EEG. Interestingly, SN echogenicity in CJD generally remains normal.

Conclusion and future directions

Structural insights using the TCS provide better insights into subtyping dementia than the TCD. Considering the limited utility of MRI in resource-limited settings, it is an invaluable noninvasive method for evaluating dementias. However, operator dependence and poor acoustic windows in some (20%) patients can limit its generalisability [2].

In 2019, the Movement Disorder Society introduced research criteria for prodromal Parkinson’s disease, incorporating likelihood ratios (LRs) derived from all available studies [5]. They developed a risk calculator that integrates multiple biomarkers, clinical, imaging, and, notably, a new addition: SN hyperechogenicity. Although TCCD is not useful as a standalone tool for identifying cognitive subtypes, its utility increases when LRs are applied systematically. Developing similar web-based tools to assess the risk of prodromal AD, VaD, FTD, and DLB. Incorporating TCD findings alongside other biomarkers could enhance diagnostic accuracy and aid in selecting patients for clinical trials.
